# Research to Advance Health and Health Care Equity for People With Communication Disabilities: An Overview of Research Symposium Papers

**DOI:** 10.1044/2022_JSLHR-22-00373

**Published:** 2022-08-29

**Authors:** Megan A. Morris

**Affiliations:** a Division of General Internal Medicine, Department of Medicine, University of Colorado Anschutz Medical Campus, Aurora, CO

## Abstract

https://doi.org/10.23641/asha.21235632

For the past 15 years, the Research Symposium, which is sponsored by the National Institute on Deafness and Other Communication Disorders (NIDCD), has been held in conjunction with the Annual Convention of the American Speech-Language-Hearing Association. In 2020, the Symposium, along with the Convention, was unfortunately cancelled due to the ongoing COVID-19 global pandemic. The year 2020 was devastating for people across our country and our world. While the disease wreaked havoc on the health of our entire country and our health care system, it devastated underserved and vulnerable populations, including persons with disability and persons who identify as Black, Latinx, Indigenous, or other people of color. The pandemic shone a light on the insidious and pervasive inequities within our health care system and society. With the rise of hate crimes against the Asian American Pacific Islander community and the senseless killing of George Floyd and subsequent Black Lives Matter movement protests, 2020 served as a potential turning point in our country and health care system in recognizing the systemic and institutional racism built into our structures, policies, laws, and practices.

The focus of the 2021 Research Symposium was Health and Health Care Equity in Communication Disorders. Despite being selected several years prior, the topic was timely. The Symposium was an opportunity to dedicate time to discussing and exploring research addressing health and health care equity experienced by persons with all types of communication disabilities. Persons with disabilities, including communication disabilities, are often an overlooked population that experiences significant disparities in health and health care outcomes. The Symposium presentations crosscut clinical populations, including hearing, speech, language, pediatric, and adult populations. Many of the speakers had disabilities themselves. The Symposium also hosted a panel of individuals who represented advocacy organizations, federal government, and policy makers who engaged in a conversation about how researchers, clinicians, advocates, and policy makers can collaboratively work together to advance equity for persons with disabilities.

This forum highlights three papers from the symposium that again represent a range of ages and populations. In the first article, Morris (2022) outlines the literature on disability health and health care disparities experienced by persons with communication disabilities. She discusses how models of disabilities contribute to how we define and perceive disability and how these models can inform how we approach and attempt to mitigate the effects of disparities. For example, she discusses how social determinants of health can contribute to the health of persons with communication disabilities, as well as how systemic ableism contributes to disparities. Using a framework for addressing disparities in health care, she outlines the current evidence on defining, understanding, and addressing disability health care disparities. She concludes the article by discussing an innovation in reducing health care disparities.

In the next article, McKee et al. (2022) outline the health and health care disparities experienced by adults with hearing loss. The authors describe system-, clinic-, provider-, and patient-level challenges and potential solutions to addressing disparities. Example solutions include standardized screening for hearing loss and related communication needs by health care teams; universal design approach to medical equipment and services; training health care providers on how to effectively communicate and care for patients with hearing loss; diversifying the health care workforce by training and hiring persons with hearing loss; and connecting patients with social supports, resources, and advocacy organizations.

In the third article, Studts (2022) describes an intervention for Deaf and Hard of Hearing (DHH) children to improve their behavioral health outcomes. She outlines how, despite having high rates of disruptive behavioral health issues, children who are DHH rarely receive services to address these issues. The proposed intervention focuses on behavioral parent training, which is an evidence-based intervention to prevent or treat disruptive behavioral problems in children and adolescents.

I hope that the Symposium and the Forum are just the beginning of a conversation. Much more research is needed to address the longstanding and persistent health and health care disparities experienced by persons with disabilities and by communities of color. To embark on this research, investment is needed in training new and seasoned researchers on how to integrate concepts of equity in their research. This will often require going outside of the communication sciences and disorders field and training or partnering with colleagues in fields such as public health, health services, and implementation sciences. Furthermore, it requires a comprehensive perspective of individuals with communication disabilities to explore how structural and institutional factors and systems contribute to disparities they experience. As outlined in their 2017–2021 strategic plan, the NIDCD has identified research addressing disparities as a priority, which will likely continue into their impending 2022–2027 strategic plan (NIDCD, 2020). Continued prioritization and investment in research to address disparities in communication disability populations is crucial to supporting and encouraging future research.

As outlined in the Communication Bill of Rights, all persons have the right to full participation in communication interactions, including the right to be treated with dignity and being addressed with respect and courtesy (Brady et al., 2016). As researchers and clinicians, we have the opportunity to uphold these rights and advance the quality of life and health of persons with communication disabilities through our research and advocacy. Now is the time to act.

## Supplementary Material

10.1044/2022_JSLHR-22-00373SMS1 Presentation Video Presentation Video
